# Diagnostic and therapeutic challenges in a case of encephalitis with neuropsychiatric manifestation

**DOI:** 10.1192/j.eurpsy.2023.1635

**Published:** 2023-07-19

**Authors:** Y. Chochev

**Affiliations:** Second psychiatric clinic, UMHATNP “St. Naum”, Sofia, Bulgaria

## Abstract

**Introduction:**

A broad spectrum of medical conditions manifest with both neurological and psychiatric symptoms. One of them is encephalitis- an inflammatory brain disease, caused by diverse etiological factors. Due to the pronounced psychopathological findings these patients frequently encounter primarily the mental health services or may appear as a part of the consultation-liaison psychiatry practice.

We present the case of a 31-year-old male firstly consulted by a psychiatrist and consequently admitted to neurological ICU. His condition developed over two-year period, developing transient psychiatric symptoms, such as anxiety, auditory hallucinations, persecutory delusions and auto-aggressive behavior; non-specific neurological findings, including pseudobulbar syndrome, oral and manual automatisms; as well as EEG paroxysmal activity. The most notable manifestations were fluctuating orientation and awareness, progressive executive function decline and cognitive impairment. In the course of the illness many psychotropic medicines had been used. The patient had shown either no improvement or low tolerance to adverse effects.

**Objectives:**

To demonstrate a challenging and provocative case of our liaison psychiatry practice, where an interdisciplinary approach was mandatory.

**Methods:**

For the needs of the psychiatric assessment a clinical interview was conducted. A neurocognitive examination via MMSE was performed. Some of the tests that took place in the neurology ward included: virological testing of blood and CSF, immunological screening for paraneoplastic syndrome and autoimmune encephalitis, MRI and EEG. The diagnosis was based on the ICD-10 criteria.

**Results:**

The mental status of the patient during the hospitalization showed no remarkable changes. The MMSE score was 22/30, correlating with a mild cognitive impairment. The neurological status fluctuated slightly over the period. Most of the tests showed none or only borderline deviations, considered nonsignificant. Some of the results were not ready prior the discharge of the patient from the hospital. After an immunomodulatory therapy there was a slight improvement in the condition of the patient.

**Conclusions:**

Based on the course of the disorder, the presence of neurological aberrations, including in the higher cortical functions, the therapeutic resistance and low adverse effects threshold, a primary psychiatric disorder was excluded. Virological, paraneoplastic and autoimmune genesis of the disorder were also ruled out. More result are expected and more examinations are needed. Postinfectious encephalitis was accepted as the most probable diagnosis.

**Disclosure of Interest:**

None Declared

